# Psychometric properties of four fear of falling rating scales in people with Parkinson’s disease

**DOI:** 10.1186/1471-2318-14-66

**Published:** 2014-05-21

**Authors:** Stina B Jonasson, Maria H Nilsson, Jan Lexell

**Affiliations:** 1 Department of Health Sciences, Lund University, PO Box 157, SE-221 00 Lund, Sweden; 2 Department of Neurology and Rehabilitation Medicine, Skåne University Hospital, SE-221 85 Lund, Sweden

**Keywords:** Parkinson disease, Psychometrics, Reliability of results, Self efficacy, Questionnaires

## Abstract

**Background:**

Fear of falling (FOF) is commonly experienced in people with Parkinson’s disease (PD). It is a predictor of recurrent falls, a barrier to physical exercise, and negatively associated with health-related quality of life. A variety of rating scales exist that assess different aspects of FOF but comprehensive head-to-head comparisons of their psychometric properties in people with PD are lacking. The aim of this study was to evaluate the psychometric properties of four FOF rating scales in people with PD. More specifically, we investigated and compared the scales’ data completeness, scaling assumptions, targeting, and reliability.

**Methods:**

The FOF rating scales were: the Falls Efficacy Scale-International (FES-I), the Swedish FES (FES(S)), the Activities-specific Balance Confidence scale (ABC), and the modified Survey of Activities and Fear of Falling in the Elderly (mSAFFE). A postal survey was administered to 174 persons with PD. Responders received a second survey after two weeks.

**Results:**

The mean (SD) age and PD duration of the 102 responders were 73 (8) and 7 (6) years, respectively. ABC had worse data completeness than the other scales (6.9 vs. 0.9–1.3% missing data). All scales had corrected item-total correlations exceeding 0.4 and showed acceptable reliabilities (Cronbach’s alpha and Intraclass Correlation Coefficient (ICC) >0.80) but only FES-I had ICC >0.90. The standard error of measurements ranged from 7% (FES-I) to 12% (FES(S)), and the smallest detectable differences ranged from 20% (FES-I) to 33% (FES(S)) of the total score ranges. ABC and FES(S) had substantially more outliers than mSAFFE and FES-I (10 and 15 vs. 3 and 4, respectively) when the two test occasions were compared.

**Conclusions:**

When assessing FOF in people with PD, the findings in the present study favoured the choice of FES-I or mSAFFE. However, FES-I was the only scale with ICC >0.90 which has been suggested as a minimum when using a scale for individual comparisons.

## Background

Parkinson’s disease (PD) is a common neurodegenerative disorder that affects balance and people with PD fall more often than age-matched healthy controls [1,2]. Fear of falling (FOF) is commonly experienced [3,4] and is a predictor of recurrent falls [5], a barrier to physical exercise [6], and is negatively associated with health-related quality of life [4]. It is therefore important to detect and follow the progress of FOF in people with PD, and FOF should be considered a crucial endpoint for interventions [4,7]. High quality rating scales assessing FOF are important in both clinical practice and research. When choosing a rating scale, one has to consider which aspects the scale should cover as well as its psychometric properties (e.g., data completeness, scaling assumptions, targeting, and reliability). Increased knowledge of the psychometric properties of FOF rating scales will facilitate the interpretation of data obtained from the scales.

A variety of rating scales exist that are said to assess different aspects of FOF [8]. The Falls Efficacy Scale-International (FES-I) assesses concerns about falling and is recommended by the Prevention of Falls Network Europe (ProFaNE) [9]. FES-I was developed by combining and modifying items from three other scales: the original FES that assesses fall-related self-efficacy [10], the Activities-specific Balance Confidence scale (ABC) that assesses balance confidence [11], and the Survey of Activities and Fear of Falling in the Elderly (SAFE) that assesses both activity level, FOF and activity restriction [12]. SAFE has later been modified into a shorter version (mSAFFE) that taps activity avoidance due to the risk of falling [13].

In a recent study, we compared the content validity of FES-I, the Swedish FES (FES(S)), ABC and mSAFFE by linking them to the International Classification of Functioning, Disability and Health [14]. The linking process showed that all four scales mainly focus on FOF in relation to mobility. The ABC almost exclusively focuses on mobility, whereas the other rating scales cover a more diverse set of activities, such as self-care (FES-I, FES(S) and mSAFFE) and activities concerning community, social, and civic life (FES-I and mSAFFE) [14].

As psychometric properties, such as validity and reliability, are sample dependent [15], specific studies are needed to determine the psychometric properties of FOF rating scales in PD. One previous Swedish study has assessed the psychometric properties of FES(S) and mSAFFE in PD with satisfying results [3]. Four studies have assessed the psychometric properties of ABC in PD [16-19]. However, three of the ABC studies have limited PD samples (n = 19 to 37) [16-18] and three are based on a limited set of psychometric analyses [16,18,19]. To our knowledge, no study has assessed the psychometric properties of FES-I in people with PD. Thus, a comprehensive head-to-head comparison of psychometric properties of FOF rating scales in people with PD is warranted and will facilitate choosing a FOF rating scale for clinical practice and research in PD.

The aim of this study was to evaluate the psychometric properties of FES-I, FES(S), ABC and mSAFFE in people with PD. More specifically, we investigated and compared the scales’ data completeness, scaling assumptions, targeting, and reliability.

## Methods

This postal survey study was sent to 174 persons with PD. It included socio-demographic and disease-related questions, as well as four FOF rating scales which were administered twice (hereafter referred to as t1 and t2), two weeks apart.

### Participants and sample size

Participants were recruited from two outpatient hospital clinics in southern Sweden and included individuals with a clinically confirmed PD diagnosis (ICD-10: G 20.9) since at least one year. Exclusion criteria were difficulties reading and writing Swedish, clinically confirmed Alzheimer’s disease, dementia, or cognitive or medical problems of a severity that were assumed to restrict giving informed consent or participating in the study. Moreover, individuals who were completely bedridden or wheelchair bound were excluded since most items in the FOF rating scales refer to walking ability. A PD specialized nurse at each of the outpatients clinics and one of the authors (SBJ) screened the medical records of all PD patients that had visited the two clinics during the past 14 months (n = 275). Fifty-nine persons (39% female) were excluded based on the exclusion criteria. Their mean (SD) age and PD duration were 76 (8) and 10 (6) years, respectively. In addition, 42 persons did not meet the inclusion criterion of a PD diagnosis of at least one year. A total of 174 possible participants remained, which was considered the final sample.

To reach a ‘good sample size’ according to recommendations for methodological quality and test-retest reliability analysis [20], we aimed at 50 to 99 participants with FOF total scores at both t1 and t2. Based on previous postal surveys in people with PD [21,22], we anticipated a response rate of approximately 65% at t1. Some additional drop outs were expected at t2, as well as some internal missing responses on the FOF rating scales.

All participants gave their written informed consent. The study was conducted in accordance with the Helsinki Declaration and was approved by the Regional Ethics Review Board in Lund, Sweden (Dnr 2013/118).

### Procedure

All 174 possible participants were mailed the following: information about the study, an informed consent form, socio-demographic and disease-related questions, the four FOF rating scales (FES-I, FES(S), ABC, and mSAFFE), and a pre-stamped return envelope. A reminder was sent after two weeks to non-responders. Responders received a second survey after about two weeks, and a reminder was sent one week later to non-responders.

The internal order of the FOF rating scales was altered to minimize the risk that the ordering affected data completeness. Four different arrangements were used so that the scales appeared an equal number of times as the first, second, third, and fourth scale. Although the order of scales was altered, the original order of items within the scales remained unchanged.

### Socio-demographic and disease-related questions

Current mobility when completing the survey at t1 and t2 was self-rated as: good (i.e., parkinsonian “on” state), good but hyperkinetic, or bad (i.e., parkinsonian “off” state). The survey at t1 included demographic questions (e.g., PD duration and living arrangements), as well as single-item questions targeting self-rated PD severity (response options: mild, moderate, or severe), self-rated general health (scored 1–5; higher = worse, inspired by the general health question in the Short Form–36) [23], activities of daily living (Parkinson’s disease Activities of Daily Living Scale; PADLS) [24], and freezing of gait (item 3 of the self-administered version [25] of the Freezing of Gait Questionnaire; FOGQsa) [26]. Both PADLS and FOGQsa have been shown to be valid and reliable in people with PD [24,25]. An open-ended question targeted the presence of diseases or health-related problems other than their PD. Dichotomous questions (Yes/No) targeted the following: dyskinesia; fluctuations with periods of increasing PD symptoms; FOF; activity avoidance due to the risk of falling; unsteadiness while walking; unsteadiness during turning in walking/standing; use of walking aid or personal support while walking indoors and outdoors, respectively; previous falls and/or near falls during the past six months. A fall was defined as “an event in which the respondent came to rest on the ground, floor, or lower level” (definition adopted from ProFaNE) [27]. A near fall was defined as “a fall initiated but arrested by support from a wall, railing, or other person, etc.” [28]. Finally, participants were asked whether they had responded to the survey themselves (with or without assistance in reading and/or writing).

### The four FOF rating scales

The FES-I assesses concerns about falling [9]. Respondents answer how concerned they are about the possibility of falling in relation to 16 different activities. Response categories are: not at all, somewhat, fairly, or very concerned (scored 1 to 4, respectively). The total score ranges from 16 to 64 (higher = worse) [9]. The Swedish translated FES-I was used in this study [29].

The FES(S) assesses fall-related self-efficacy [30]. Respondents answer how confident they are in performing 13 different activities without falling. Response categories range from 0 (not confident at all) to 10 (completely confident). The total score ranges from 0 to 130 (higher = better) [30].

The ABC assesses balance confidence [11]. Respondents answer how confident they are that they would not lose their balance or become unsteady when performing 16 different activities [11]. In this study, a Swedish translated and culturally adapted version of the ABC was used. The cultural adaptation implies that items related to stepping onto or off escalators are changed to traveling by bus (L. Lundin-Olsson, unpublished material, written personal communication, June 20, 2012). Response categories range from 0 (no confidence) to 10 (completely confident). The total score is the mean value of the 16 items, transformed into percentage, i.e., ranges from 0 to 100% (higher = better).

The mSAFFE assesses activity avoidance due to the risk of falling in relation to 17 different activities [13]. Response categories are: never, sometimes, or always avoid (scored 1 to 3, respectively). The total score ranges from 17 to 51 (higher = worse) [13]. The Swedish translated mSAFFE was used in this study (L. Lundin-Olsson, unpublished material, written personal communication, June 20, 2012).

### Analyses

The analyses were performed using the IBM SPSS Statistics 21.0 software and were based on four parts: i) data completeness, ii) scaling assumptions, iii) targeting, and iv) reliability. Data completeness and reliability (except Cronbach’s alpha) were based on data from both t1 and t2. Scaling assumptions, targeting and Cronbach’s alpha were based on t1 data only. The relationships between the rating scales were determined by calculating the Pearson’s correlation coefficients (*r*) between the scales, based on t1 data.

#### Data completeness

Data completeness of the four rating scales was determined by calculating the percentage of missing data for items and total scores [15,31]. No imputation was done, i.e., a total score required absence of any missing item responses.

#### Scaling assumptions

Scaling assumptions were explored to examine the legitimacy of summing item scores to generate total scale scores, according to a series of criteria [15,31]. That is, mean scores, SDs, and distribution of item response option frequency should be roughly parallel across items. Also, corrected item-total correlations should exceed 0.4, indicating that items measure the same underlying construct and contain a similar proportion of information concerning FOF [15,31].

#### Targeting

Targeting refers to whether the rating scales’ score distributions can adequately represent the true level of FOF in the sample [15]. This was evaluated by studying the rating scales’ score distribution, skewness, and floor and ceiling effects. Mean total scores should be close to the scales’ midpoint, total scores range the full span, skewness less than ±1 [15,32], and floor and ceiling effects (the percentage respondents receiving the minimum and maximum possible scores, respectively) should not exceed 15–20% [15,33].

#### Reliability

Reliability is a measure of the random error associated with scale scores and the reproducibility of scores [15]. This was assessed in several ways. The internal consistency was examined by means of Cronbach’s alpha [15]. The test-retest reliability was studied in terms of one-way random, single measures Intraclass Correlation Coefficient (ICC_1,1_) with absolute agreement definition of concordance [34]. Cronbach’s alpha and ICC >0.75 or >0.80 are considered acceptable for group level [35,36], while ICC >0.90 has been suggested as a minimum when using scales for individual comparisons [36,37]. The standard error of measurement (SEM) was calculated using the formula ${\mathrm{SD}}_{\mathrm{baseline}}\times \sqrt{1-\mathrm{reliability}}$[38]. The smallest detectable difference (SDD) was calculated using the formula $\mathrm{SEM}\times 1.96\times \sqrt{2}$[39]. Due to differences in scoring ranges between the scales, SEM and SDD values were also expressed as percentages of the possible scoring ranges, to facilitate comparisons.

The mean difference (đ) in scale scores between t1 and t2 and the 95% CI around đ were calculated. If the 95% CI includes 0, there are no systematic differences between t1 and t2 [40]. The number of outliers for each rating scale was calculated (an outliers was defined as a participant with differences between t1 and t2 outside the first or third quartile ± 1.5 × interquartile range) [41]. Finally, test-retest data were plotted and visually inspected in the form of Bland-Altman graphs (the individual differences between t1 and t2 were plotted against the individual mean of t1 and t2) [40]. Since these graphs did not contribute any additional information than the numerical analyses, they are not presented here.

## Results

Of the 174 possible participants, 63 persons did not respond and 6 explicitly expressed that they did not want to participate; they (n = 69; 54% women) had a mean (SD) age of 77 (9) years. One hundred and five persons returned the first postal survey, but three surveys were not answered by the person with PD and were therefore excluded. This resulted in 102 included participants and a conservative response rate of 59%. Ninety-seven persons responded to the second survey (t2). Basic demographic data and participants characteristics are presented in Table 1. A majority (n = 60) of the participants stated that they had one or more disease or health-related problem, apart from their PD. The most common problems were cardiovascular (n = 22) and musculoskeletal (n = 22). Current mobility at t1 was rated as good (i.e., parkinsonian “on” state) by 48 participants, good but hyperkinetic by 17, and bad (i.e., parkinsonian “off” state) by 35 participants (2 missing responses). Corresponding mobility ratings at t2 were: good (“on”) 52 participants, good but hyperkinetic 15, and bad (“off”) 23 participants (7 missing responses).

**Table 1:** Participants’ characteristics

Gender (women)	43/102
Age (years), mean (SD), min-max	73 (8), 52–91^d^
Parkinson duration (years), mean (SD), min-max	7 (6), 1–30^e^
Self-rated Parkinson severity	
Mild	24/98
Moderate	61/98
Severe	13/98
Self-rated general health, median (first-third quartile)^a^	4 (3–4)^d^
Dyskinesia (yes)	40/101
Fluctuations with periods of increasing parkinsonian symptoms (yes)	52/97
Living alone (yes)	28/101
Fear of falling (yes)	56/102
Activity avoidance due to risk of falling (yes)	54/101
Falls past 6 months (yes)	36/102
Near falls past 6 months (yes)	56/102
Unsteady during walking (yes)	56/97
Unsteady during turning in walking/standing (yes)	64/96
Use of walking aid indoors/outdoors, respectively (yes)	25/96 and 43/97
Need personal support during walking indoors and outdoors, respectively (yes)	4/97 and 22/95
Need help from others in daily activities (yes)^b^	19/92
Freezing of gait (yes)^c^	44/93

Data are n/total unless otherwise stated.

^a^Possible scoring range 1–5, higher = worse.

^b^Parkinson’s disease Activities of Daily Living Scale. Dichotomized: “no difficulties” and “mild difficulties” are counted as “no”. “Moderate difficulties”, “high levels of difficulties” and “extreme difficulties” are counted as “yes”.

^c^Item 3 of the self-administered Freezing of Gait Questionnaire. Dichotomized: all response options but “never” are counted as “yes”.

^d^n = 102.

^e^n = 98.

### Relationship between the scales

The correlations (*r*) between the four FOF rating scales ranged from 0.80 to 0.93 (*P* < 0.001); the weakest correlation was found between mSAFFE and ABC and the strongest between mSAFFE and FES-I.

### Data completeness

One of the 102 participants left FES-I completely blank and another person left both FES(S) and ABC blank. Four additional persons misunderstood the ABC: three persons responded by writing “X” instead of specifying a digit after the items, and the fourth person supplied double digits on each item, resulting in uninterpretable responses. The number of participants that obtained a total score was: ABC, n = 82; mSAFFE, n = 86; FES(S), n = 90; and FES-I, n = 92. The overall mean of missing responses were: FES-I, 0.9%; FES(S), 1.0%; mSAFFE, 1.3%; and ABC, 6.9% (those that left the scales completely blank are not included in these numbers). The number of participants that obtained a total score at t2 was: ABC, n = 79; FES(S), n = 85; mSAFFE, n = 86; and FES-I, n = 90.

### Scaling assumptions

Item means and SDs, respectively, were roughly parallel for most items in each of the FOF scales. Some items of FES-I, ABC and mSAFFE had a larger proportion of participants that chose the worse response options, resulting in worse mean scores (i.e., more difficult items). These were: FES-I items 11 (Walk on slippery surface), 14 (Walk on uneven surface) and 15 (Walk up/down a slope), ABC items 6 (Stand on chair to reach) and 16 (Walk on icy sidewalks), and mSAFFE item 8 (Go out when it is slippery) (Tables 2, 3, 4, 5). A larger proportion of responders chose the best response option for FES-I item 3 (Preparing simple meals) and mSAFFE items 4 (Go to the doctor/dentist), 6 (Take a shower) and 12 (Walk around indoors) (data available on request). All four scales had corrected item-total correlations exceeding 0.4 (Tables 2, 3, 4, 5).

**Table 2:** Scoring distribution and data completeness of Falls Efficacy Scale-International (time 1 data)

**Items**	**Activity**	**Mean (SD) n = 101**^**a**^	**Missing or invalid responses**	**Corrected item-total correlations n = 92**^**b**^
1.	Cleaning the house	2.0 (1.1)	2	0.85
2.	Getting dressed or undressed	1.5 (0.8)	1	0.65
3.	Preparing simple meals	1.4 (0.8)	2	0.61
4.	Taking a bath or shower	1.6 (1.0)	-	0.76
5.	Buying some groceries	1.7 (1.1)	1	0.82
6.	Getting in or out of a chair	1.7 (0.8)	2	0.59
7.	Climbing stairs	2.0 (1.0)	1	0.85
8.	Walking around in the neighbourhood	1.7 (0.9)	-	0.79
9.	Reaching for something above your head or on the ground	2.0 (1.0)	1	0.77
10.	Answering the telephone before it stops ringing	1.7 (0.9)	2	0.75
11.	Walking on a slippery surface	2.7 (1.0)	-	0.75
12.	Visiting acquaintances, friends or relatives	1.7 (0.9)	1	0.78
13.	Walking in crowds	1.9 (1.0)	-	0.74
14.	Walking on an uneven surface	2.3 (1.1)	1	0.81
15.	Walking up or down a slope	2.3 (1.1)	-	0.76
16.	Participating in a social event	1.7 (0.9)	-	0.72
Total score (n = 92)		29.6 (12.0)		
Min-Max		16–59		
Skewness (SE)		0.72 (0.25)		
Floor/ceiling effects (%)		9.8/0		

SE = standard error.

^a^One person left the questionnaire blank. This person is not included in the missing data.

^b^Nine persons did not answer all 16 items and did therefore not receive a total score.

Possible item score range 1–4, possible total score range 16–64, higher = worse.

**Table 3:** Scoring distribution and data completeness of the Swedish Falls Efficacy Scale (time 1 data)

**Items**	**Activity**	**Mean (SD) n = 101**^**a**^	**Missing or invalid responses**	**Corrected item-total correlations n = 90**^**b**^
1.	Get in and out of bed	7.2 (2.7)	1	0.88
2.	Go to the toilet	7.5 (2.7)	1	0.94
3.	Wash yourself	8.0 (2.6)	1	0.79
4.	Get in and out of a chair	7.2 (2.7)	-	0.82
5.	Get dressed and undressed	7.3 (2.9)	1	0.89
6.	Take a bath or a shower	7.1 (3.3)	-	0.89
7.	Go up and down stairs	6.4 (3.4)	4	0.86
8.	Walk around the neighbourhood	6.9 (3.4)	3	0.93
9.	Reach into cupboards/closets	6.9 (3.4)	-	0.84
10.	Clean the apartment	6.6 (3.6)	-	0.90
11.	Prepare a meal that does not require carrying hot or heavy objects	6.9 (3.4)	1	0.87
12.	Hurrying up to answer the telephone	6.5 (3.4)	-	0.82
13.	Simple shopping	6.7 (3.6)	1	0.87
Total score (n = 90)		93.5 (36.4)		
Min-Max		11–130		
Skewness (SE)		–0.64 (0.25)		
Floor/ceiling effects (%)		0/17.8		

SE = standard error.

^a^One person left the questionnaire blank. This person is not included in the missing data.

^b^Eleven persons did not answer all 13 items and did therefore not receive a total score.

Possible item score range 0–10, possible total score range 0–130, higher = better.

**Table 4:** Scoring distribution and data completeness of Activities-specific Balance Confidence scale (time 1 data)

**Items**	**Activity**	**Mean (SD) n = 97**^**a**^	**Missing or invalid responses**^**b**^	**Corrected item-total correlations n = 82**^**c**^
1.	Walk around the house	7.2 (2.7)	5	0.84
2.	Walk up or down stairs	6.0 (3.4)	6	0.90
3.	Bend over and pick up a shoe from the floor	6.4 (3.0)	5	0.86
4.	Reach for a small can off a shelf at eye level	7.5 (2.8)	6	0.80
5.	Stand on your tiptoes and reach for something above your head	6.1 (3.3)	6	0.83
6.	Stand on a chair and reach for something	4.4 (3.8)	9	0.80
7.	Sweep or vacuum the floor	6.3 (3.7)	7	0.87
8.	Walk to a taxi that is waiting by the sidewalk	6.9 (3.3)	6	0.89
9.	Get into or out of a car	6.7 (3.0)	5	0.88
10.	Cross a street	6.6 (3.5)	8	0.90
11.	Step onto or off a curb	6.8 (3.3)	5	0.90
12.	Walk on a street where people are rapidly passing	6.8 (3.2)	6	0.91
13.	Others bump into you as you walk on the street	5.9 (3.5)	5	0.90
14.	Travel by bus *without* a bag of groceries	6.6 (3.8)	13	0.89
15.	Travel by bus *with* a bag of groceries	6.1 (3.8)	13	0.90
16.	Walk on icy sidewalks	3.7 (3.5)	6	0.75
Total score (n = 82)		62.4 (29.6)		
Min-Max		1–100		
Skewness (SE)		–0.45 (0.27)		
Floor/ceiling effects (%)		0/4.9		

SE = standard error.

^a^One person left the questionnaire blank and four persons misunderstood the entire scale.

^b^Missing data includes the four persons who misunderstood the scale, but not the person who left it blank.

^c^Fifteen persons did not answer all 16 items and did therefore not receive a total score.

Possible item score range 0–10, possible total score range 0–100, higher = better.

**Table 5:** Scoring distribution and data completeness of modified Survey of Activities and Fear of Falling in the Elderly (time 1 data)

**Items**	**Activity**	**Mean (SD) n = 102**	**Missing or invalid responses**	**Corrected item-total correlations n = 86**^**a**^
1.	Walk to the store and shop	1.7 (0.8)	3	0.87
2.	Clean your house	1.6 (0.7)	1	0.77
3.	Prepare simple meals	1.3 (0.5)	1	0.58
4.	Go to the doctor or dentist	1.2 (0.4)	1	0.59
5.	Take a bath	1.5 (0.7)	7	0.61
6.	Take a shower	1.3 (0.5)	-	0.54
7.	Go for a walk	1.5 (0.6)	-	0.75
8.	Go out when it is slippery	2.2 (0.7)	-	0.64
9.	Visit a friend or relative	1.4 (0.6)	3	0.81
10.	Walk to a place with crowds	1.8 (0.7)	1	0.76
11.	Climb stairs	1.6 (0.7)	-	0.78
12.	Walk around indoors	1.1 (0.3)	1	0.57
13.	Walk a kilometer	1.8 (0.8)	1	0.74
14.	Bend down to pick up something	1.6 (0.6)	-	0.68
15.	Travel by public transport	1.7 (0.8)	3	0.71
16.	Attend a social event or party	1.5 (0.6)	-	0.68
17.	Reach for something above your head	1.7 (0.7)	-	0.60
Total score (n = 86)		26.0 (7.9)		
Min-Max		17–47		
Skewness (SE)		0.80 (0.26)		
Floor/ceiling effects (%)		10.5/0		

SE = standard error.

^a^Sixteen persons did not answer all 17 items and did therefore not receive a total score.

Possible item score range 1–3, possible total score range 17–51, higher = worse.

### Targeting

All four scales spanned almost the full range of possible scale scores and the scales’ mean scores were close to the scales’ midpoints (i.e., FES-I, 40; FES(S), 65; ABC, 50; mSAFFE, 34). Skewness was < ±1, and floor and ceiling effects were <20% for all four scales (Tables 2, 3, 4, 5).

### Reliability

The mean time between responses to the first and the second survey was 16.7 (SD 3.8, min-max 13–38) days. Reliability coefficients, SEM and SDD values for the four FOF scales are presented in Table 6. All scales had Cronbach’s alpha >0.90 and ICC >0.80, and one (FES-I) had ICC >0.90. The đ was close to 0 with CI including 0 for all four scales. There were 3 outliers in mSAFFE, 4 in FES-I, 10 in ABC, and 15 in FES(S).

**Table 6:** Reliability of the four fear of falling rating scales

**Rating scale**	**Cronbach’s alpha (time 1)**	**ICC (95% CI)**	**đ(95% CI)**^**a**^	**SEM (% of possible scoring range)**^**b**^	**SDD (% of possible scoring range)**^**c**^
FES-I	0.96	0.92 (0.88–0.95) n = 81	–0.05 (–1.09–0.99)	3.4 (7)	9.6 (20)
FES(S)	0.98	0.82 (0.73–0.88) n = 76	–2.17 (–6.95–2.61)	15.4 (12)	42.7 (33)
ABC	0.98	0.86 (0.79–0.91) n = 68	–0.04 (–3.68–3.60)	11.0 (11)	30.5 (30)
mSAFFE	0.94	0.85 (0.78–0.90) n = 76	0.76 (–0.19–1.72)	3.0 (9)	8.4 (24)

FES-I = Falls Efficacy Scale-International, FES(S) = Swedish Falls Efficacy Scale, ABC = Activities-specific Balance Confidence scale, mSAFFE = modified Survey of Activities and Fear of Falling in the Elderly, ICC = intraclass correlation coefficient (one-way random model, absolute agreement, single measures), SEM = standard error of measurement, SDD = smallest detectable difference.

^a^đ defined as mean difference in scale scores (*time* 2 − *time* 1).

^b^SEM defined as ${\mathrm{SD}}_{\mathrm{baseline}}\times \sqrt{1-\mathrm{ICC}}$.

^c^SDD defined as $\mathrm{SEM}\times 1.96\times \sqrt{2}$.

## Discussion

This is the first comprehensive comparison of the psychometric properties of four commonly used FOF scales in people with PD. Our main findings were: ABC had markedly worse data completeness than the other scales, all scales showed acceptable reliability (Cronbach’s alpha and ICC >0.80) but only FES-I had ICC >0.90, and FES(S) and ABC had substantially more outliers than mSAFFE and FES-I when comparing t1 and t2.

Our sample consisted of more males than females, which is in agreement with prevalence studies of PD [42]. The mean age and PD duration were 73 and 7 years, respectively, which correspond well with a previously reported mean age at symptom onset of 62 to 70 years [43]. Our sample contained fewer fallers than previous studies [3,44], whereas the prevalence of FOF in our sample (55%) was within a previously reported range (38–59%) [3,4]. Self-reported PD severity ranged from mild to severe. The present sample thus seems fairly representative, although it needs to be noted that those with severe cognitive or medical problems were excluded.

### Relationship between the scales

The four FOF rating scales correlated ≥0.80, which is not surprising since the content is similar [14]. However, the scales are said to assess different aspects of FOF, i.e., concerns about falling, fall-related self-efficacy, balance confidence and activity avoidance due to the risk of falling [9,11,13,30]. Previous studies have stated that these constructs are not interchangeable and that scale selection should be based on the specific construct of interest [3,8]. Thus, more studies are needed to confirm the relationships between the different FOF scales.

### Data completeness

ABC had a substantially higher proportion of missing data than the other scales (6.9 vs. 0.9–1.3%). Four persons completely misunderstood the ABC, implying that the instructions need to be clarified. It should, however, be noted that the Swedish version of the ABC was used in this study and the instructions might be perceived as more clear in the original ABC. To our knowledge, no previous study has presented data completeness for ABC in people with PD or other samples.

The percentage of missing data was highest (12.9%) for ABC items 14 and 15. These are the items that are culturally adapted in the Swedish version (changed from stepping on/off escalators into traveling by bus, L. Lundin-Olsson, written personal communication, June 20, 2012). The high number of missing data suggests that these items are difficult to understand or irrelevant to the participants [31]. In fact, three participants had written supplementary comments, stating that they did not travel by bus. An additional 19 participants stated that they always avoided traveling by public transport according to item 15 of mSAFFE. While the original ABC includes instructions on how to respond to activities that the respondent does not engage in, the Swedish translated ABC does not. This might explain the high number of missing responses in these items. However, even if these two items are removed, missing data remains higher for ABC than for the other scales (6.0% vs. 0.9–1.3%).

### Scaling assumptions

While FES(S) items were roughly parallel, this was not the case for the other three scales. These findings are not unexpected since the various items within the scales are of different difficulty level. Moreover, items that were rated as more difficult by our sample have been rated as more difficult in previous PD studies as well as in older healthy populations [3,9,11,17]. One could argue that some variation in item difficulty levels is preferable, since this result in a scale that is able to assess FOF in individuals with both low and high levels of FOF. Although classic test theory states that items within a scale should be “roughly parallel” to allow for a summed total score [15,31], no guidelines exist that describe how rigid this judgement should be. Previous studies using the FOF scales studied here have, in fact, all used regular total scores [3,9,11,13,17,18,45].

### Targeting

All four scales seem fairly well targeted and met the criterion of floor and ceiling effects below 20% [15]. FES(S) had 17.8% ceiling effect, which is higher than the other scales (4.9–10.5% floor/ceiling effect). A previous PD study found a lower ceiling effect of FES(S) (10.1–10.6%), but a higher floor effect of mSAFFE (18.3–19.4% vs. 10.5% in our study) [3]. No previous study has presented data on floor and ceiling effects on FES-I and ABC in people with PD.

### Reliability

All four scales had high internal consistency (Cronbach’s alpha >0.90) and acceptable test-retest reliability (ICC >0.80) [35,36]. However, only FES-I had an ICC >0.90, which has been suggested as a minimum when using scales for individual comparisons [36,37]. In comparison with previous reliability studies in PD, our results of internal consistency were consistent with previous studies [3,16-19]. Test-retest reliability of FES(S) was lower than previously reported (ICC = 0.82 vs. ICC = 0.87) [3]. The situation was similar for mSAFFE (0.85 vs. 0.92) [3]. The ICC of ABC in the current study was in between the results of the two previous ABC studies that assessed test-retest reliability (0.86 vs. 0.79 and 0.94) [17,18]. These differences are likely to appear as psychometric properties are sample dependent [15].

SEM% for the four rating scales varied from 7 to 12%. This implies that a change in a mean score greater than 7 to 12% of the possible scoring range indicates a “real” change (above measurement error), when assessing FOF for a group of people with PD [38]. SDD% were 20 to 33%, indicating that the smallest change in an individual’s FOF score that can be interpreted as a “real” change (above measurement error) should exceed 20 to 33% of the possible scoring ranges [39]. FES-I had the lowest SEM% and SDD%, where a difference of at least 4 and 10 points indicated a “real” change on a group and individual level, respectively.

### Study limitations

There is a variety of FOF scales [8], and it needs to be acknowledged that this psychometric comparison is not fully comprehensive since only four Swedish translated scales were included. We selected FES-I because it is recommended by ProFaNE [9], and its forerunners (or adaptations of them) since they are commonly used. More studies of other FOF rating scales are needed, as well as cross-national comparisons, to establish which rating scale that is the best of *all* available FOF scales.

The postal survey study design means that all scales were self-administered, and it needs to be underlined that the present findings may not apply if the scales are administered as an interview. Furthermore, the cross-sectional design does not enable us to determine either the responsiveness of the FOF scales, nor the minimal important differences. However, it has been argued that SEM is a reasonable approximation of the minimal important difference [46].

## Conclusions

All four FOF scales showed acceptable internal consistency and test-retest reliability. ABC revealed insufficiencies in terms of data completeness, and ABC and FES(S) had many outliers when comparing t1 and t2. When assessing FOF in people with PD, the findings in the present study favoured the choice of FES-I or mSAFFE. However, FES-I was the only scale with ICC >0.9, which has been suggested when using a scale for individual comparisons.

## Competing interests

The authors declare that they have no competing interest.

## Authors’ contributions

SBJ, MHN and JL conceived and designed the study. SBJ performed data collection, analysed the data and drafted the initial manuscript. All authors participated in writing (and approved) the final version of the manuscript.

## Pre-publication history

The pre-publication history for this paper can be accessed here:

http://www.biomedcentral.com/1471-2318/14/66/prepub

## References

[B1] Suarez H Geisinger D Suarez A Carrera X Buzo R Amorin I Postural control and sensory perception in patients with Parkinson’s disease Acta Otolaryngol 2009 14 354 360 10.1080/00016480802495446 19021071

[B2] Mak MK Pang MY Parkinsonian single fallers versus recurrent fallers: different fall characteristics and clinical features J Neurol 2010 14 1543 1551 10.1007/s00415-010-5573-9 20449601

[B3] Nilsson MH Drake AM Hagell P Assessment of fall-related self-efficacy and activity avoidance in people with Parkinson’s disease BMC Geriatr 2010 14 78 10.1186/1471-2318-10-78 20973974 PMC2984450

[B4] Grimbergen YA Schrag A Mazibrada G Borm GF Bloem BR Impact of falls and fear of falling on health-related quality of life in patients with Parkinson’s disease J Parkinsons Dis 2013 14 409 413 23948987 10.3233/JPD-120113

[B5] Mak MK Pang MY Fear of falling is independently associated with recurrent falls in patients with Parkinson’s disease: a 1-year prospective study J Neurol 2009 14 1689 1695 10.1007/s00415-009-5184-5 19479166

[B6] Ellis T Boudreau JK DeAngelis TR Brown LE Cavanaugh JT Earhart GM Ford MP Foreman KB Dibble LE Barriers to exercise in people with Parkinson disease Phys Ther 2013 14 628 636 10.2522/ptj.20120279 23288910 PMC3641403

[B7] Nilsson MH Hariz GM Iwarsson S Hagell P Walking ability is a major contributor to fear of falling in people with Parkinson’s disease: implications for rehabilitation Parkinsons Dis 2012 14 713236 21941686 10.1155/2012/713236 PMC3175698

[B8] Moore DS Ellis R Measurement of fall-related psychological constructs among independent-living older adults: a review of the research literature Aging Ment Health 2008 14 684 699 10.1080/13607860802148855 19023720

[B9] Yardley L Beyer N Hauer K Kempen G Piot-Ziegler C Todd C Development and initial validation of the Falls Efficacy Scale-International (FES-I) Age Ageing 2005 14 614 619 10.1093/ageing/afi196 16267188

[B10] Tinetti ME Richman D Powell L Falls efficacy as a measure of fear of falling J Gerontol 1990 14 P239 P243 10.1093/geronj/45.6.P239 2229948

[B11] Powell LE Myers AM The activities-specific balance confidence (ABC) scale J Gerontol A Biol Sci Med Sci 1995 14 M28 M34 10.1093/gerona/50A.1.M28 7814786

[B12] Lachman ME Howland J Tennstedt S Jette A Assmann S Peterson EW Fear of falling and activity restriction: the survey of activities and fear of falling in the elderly (SAFE) J Gerontol B Psychol Sci Soc Sci 1998 14 P43 P50 9469171 10.1093/geronb/53b.1.p43

[B13] Yardley L Smith H A prospective study of the relationship between feared consequences of falling and avoidance of activity in community-living older people Gerontologist 2002 14 17 23 10.1093/geront/42.1.17 11815695

[B14] Bladh S Nilsson MH Carlsson G Lexell J Content analysis of 4 fear of falling rating scales by linking to the international classification of functioning, disability and health PM R 2013 14 573 582 e571 10.1016/j.pmrj.2013.01.006 23466949

[B15] Hobart J Cano S Improving the evaluation of therapeutic interventions in multiple sclerosis: the role of new psychometric methods Health Technol Assess 2009 14 1 177 10.3310/hta13120 19216837

[B16] Peretz C Herman T Hausdorff JM Giladi N Assessing fear of falling: can a short version of the activities-specific balance confidence scale be useful? Mov Disord 2006 14 2101 2105 10.1002/mds.21113 16991140

[B17] Dal Bello-Haas V Klassen L Sheppard MS Metcalfe A Psychometric properties of activity, self-efficacy, and quality-of-life measures in individuals with Parkinson disease Physiother Can 2011 14 47 57 10.3138/ptc.2009-08 22210979 PMC3024195

[B18] Steffen T Seney M Test-retest reliability and minimal detectable change on balance and ambulation tests, the 36-item short-form health survey, and the unified Parkinson disease rating scale in people with parkinsonism Phys Ther 2008 14 733 746 10.2522/ptj.20070214 18356292

[B19] Lohnes CA Earhart GM External validation of abbreviated versions of the activities-specific balance confidence scale in Parkinson’s disease Mov Disord 2010 14 485 489 10.1002/mds.22924 20014055 PMC2873031

[B20] Terwee CB Mokkink LB Knol DL Ostelo RW Bouter LM de Vet HC Rating the methodological quality in systematic reviews of studies on measurement properties: a scoring system for the COSMIN checklist Qual Life Res 2012 14 651 657 10.1007/s11136-011-9960-1 21732199 PMC3323819

[B21] Bladh S Nilsson MH Hariz GM Westergren A Hobart J Hagell P Psychometric performance of a generic walking scale (Walk-12G) in multiple sclerosis and Parkinson’s disease J Neurol 2012 14 729 738 10.1007/s00415-011-6254-z 21956376

[B22] Nilsson MH Bladh S Hagell P Fatigue in Parkinson’s disease: measurement properties of a generic and a condition-specific rating scale J Pain Symptom Manage 2013 14 737 746 10.1016/j.jpainsymman.2012.11.004 23507131

[B23] Ware JE Jr Sherbourne CD The MOS 36-item short-form health survey (SF-36) I. Conceptual framework and item selection Med Care 1992 14 473 483 10.1097/00005650-199206000-00002 1593914

[B24] Hobson JP Edwards NI Meara RJ The Parkinson’s disease activities of daily living scale: a new simple and brief subjective measure of disability in Parkinson’s disease Clin Rehabil 2001 14 241 246 10.1191/026921501666767060 11386393

[B25] Nilsson MH Hariz GM Wictorin K Miller M Forsgren L Hagell P Development and testing of a self administered version of the freezing of gait questionnaire BMC Neurol 2010 14 85 10.1186/1471-2377-10-85 20863392 PMC2955563

[B26] Giladi N Shabtai H Simon ES Biran S Tal J Korczyn AD Construction of freezing of gait questionnaire for patients with Parkinsonism Parkinsonism Relat Disord 2000 14 165 170 10.1016/S1353-8020(99)00062-0 10817956

[B27] Lamb SE Jorstad-Stein EC Hauer K Becker C Development of a common outcome data set for fall injury prevention trials: the prevention of falls network Europe consensus J Am Geriatr Soc 2005 14 1618 1622 10.1111/j.1532-5415.2005.53455.x 16137297

[B28] Gray P Hildebrand K Fall risk factors in Parkinson’s disease J Neurosci Nurs 2000 14 222 228 10.1097/01376517-200008000-00006 10994536

[B29] Nordell E Andreasson M Gall K Thorngren K-G Evaluating the Swedish version of the falls efficacy scale-international (FES-I) Adv Physiother 2009 14 81 87 10.1080/14038190802318986

[B30] Hellstrom K Lindmark B Fear of falling in patients with stroke: a reliability study Clin Rehabil 1999 14 509 517 10.1191/026921599677784567 10588538

[B31] Ware JE Jr Gandek B Methods for testing data quality, scaling assumptions, and reliability: the IQOLA Project approach. International Quality of Life Assessment J Clin Epidemiol 1998 14 945 952 10.1016/S0895-4356(98)00085-7 9817111

[B32] Hobart JC Riazi A Lamping DL Fitzpatrick R Thompson AJ Improving the evaluation of therapeutic interventions in multiple sclerosis: development of a patient-based measure of outcome Health Technol Assess 2004 14 iii 1–48 14982653 10.3310/hta8090

[B33] McHorney CA Tarlov AR Individual-patient monitoring in clinical practice: are available health status surveys adequate? Qual Life Res 1995 14 293 307 10.1007/BF01593882 7550178

[B34] Schuck P Assessing reproducibility for interval data in health-related quality of life questionnaires: which coefficient should be used? Qual Life Res 2004 14 571 586 15130022 10.1023/B:QURE.0000021318.92272.2a

[B35] Shrout PE Fleiss JL Intraclass correlations: uses in assessing rater reliability Psychol Bull 1979 14 420 428 18839484 10.1037//0033-2909.86.2.420

[B36] Nunnally JC Bernstein IH Psychometric theory 1994 3 New York: McGraw-Hill

[B37] Scientific Advisory Committee of the Medical Outcomes Trust Assessing health status and quality-of-life instruments: attributes and review criteria Qual Life Res 2002 14 193 205 10.1023/A:1015291021312 12074258

[B38] Streiner DL Norman GR Health measurement scales: a practical guide to their development and use 2008 4 Oxford; New York: Oxford University Press

[B39] Terwee CB Bot SD de Boer MR van der Windt DA Knol DL Dekker J Bouter LM de Vet HC Quality criteria were proposed for measurement properties of health status questionnaires J Clin Epidemiol 2007 14 34 42 10.1016/j.jclinepi.2006.03.012 17161752

[B40] Lexell JE Downham DY How to assess the reliability of measurements in rehabilitation Am J Phys Med Rehabil 2005 14 719 723 10.1097/01.phm.0000176452.17771.20 16141752

[B41] Norman GR Streiner DL Biostatistics: the bare essentials 2008 3 Shelton, Conn: People’s Medical Pub. House

[B42] Wirdefeldt K Adami HO Cole P Trichopoulos D Mandel J Epidemiology and etiology of Parkinson’s disease: a review of the evidence Eur J Epidemiol 2011 14 Suppl 1 S1 S58 21626386 10.1007/s10654-011-9581-6

[B43] Muangpaisan W Mathews A Hori H Seidel D A systematic review of the worldwide prevalence and incidence of Parkinson’s disease J Med Assoc Thai 2011 14 749 755 21696087

[B44] Bloem BR Hausdorff JM Visser JE Giladi N Falls and freezing of gait in Parkinson’s disease: a review of two interconnected, episodic phenomena Mov Disord 2004 14 871 884 10.1002/mds.20115 15300651

[B45] Allen NE Canning CG Sherrington C Lord SR Latt MD Close JC O’Rourke SD Murray SM Fung VS The effects of an exercise program on fall risk factors in people with Parkinson’s disease: a randomized controlled trial Mov Disord 2010 14 1217 1225 10.1002/mds.23082 20629134

[B46] King MT A point of minimal important difference (MID): a critique of terminology and methods Expert Rev Pharmacoecon Outcomes Res 2011 14 171 184 10.1586/erp.11.9 21476819

